# Microscopic and Spectroscopic Imaging and Thermal Analysis of Acrylates, Silicones and Active Pharmaceutical Ingredients in Adhesive Transdermal Patches

**DOI:** 10.3390/polym14142888

**Published:** 2022-07-16

**Authors:** Barbara Mikolaszek, Marzena Jamrógiewicz, Krystyna Mojsiewicz-Pieńkowska, Małgorzata Sznitowska

**Affiliations:** 1 Department of Pharmaceutical Technology, Faculty of Pharmacy, Medical University of Gdańsk, 80-416 Gdańsk, Poland; barbara.mikolaszek@gumed.edu.pl; 2 Department of Physical Chemistry, Faculty of Pharmacy, Medical University of Gdańsk, 80-416 Gdańsk, Poland; marzena.jamrogiewicz@gumed.edu.pl (M.J.); krystyna.pienkowska@gumed.edu.pl (K.M.-P.)

**Keywords:** adhesive patches, silicones, acrylates, indomethacin, testosterone, cytisine, infrared spectroscopy (FTIR), Raman spectroscopy, microscopy, differential scanning calorimetry (DSC)

## Abstract

Dermal or transdermal patches are increasingly becoming a noteworthy alternative as carriers for active pharmaceutical ingredients (APIs), which makes their detailed physicochemical evaluation essential for pharmaceutical development. This paper demonstrates mid-infrared (FTIR) and Raman spectroscopy with complementary microscopic methods (SEM, optical and confocal Raman microscopy) and differential scanning calorimetry (DSC) as tools for the identification of the state of model API (testosterone TST, cytisine CYT or indomethacin IND) in selected adhesive matrices. Among the employed spectroscopic techniques, FTIR and Raman may be used not only as standard methods for API identification in the matrix, but also as a means of distinguishing commercially available polymeric materials of a similar chemical structures. A novel approach for the preparation of adhesive polymers for the FTIR analysis was introduced. In silicone matrices, all three APIs were suspended, whereas in the case of the acrylic PSA, Raman microscopy confirmed that only IND was dissolved in all three acrylic matrices, and the dissolved fraction of the CYT differed depending on the matrix type. Moreover, the recrystallization of TST was observed in one of the acrylates. Interestingly, a DSC analysis of the acrylic patches did not confirm the presence of the API even if the microscopic images showed suspended particles.

## 1. Introduction

Over the last 30 years, polymeric patches have been used for the transdermal delivery of a variety of active substances with the aim of achieving a local or systemic effect. Active pharmaceutical ingredients (APIs) such as nicotine, testosterone or diclofenac sodium are presented in such application forms [1,2].

Sufficient skin adhesion is assured by pressure-sensitive adhesive polymers (PSAs) which adhere easily to the skin surface under light pressure and are capable of staying in the area of application for up to several days, while at the same time, the patch can be easily and painlessly removed without damaging the skin [3,4]. Among the most commonly used PSA polymers, one should mention polyisobutylene and acrylates, with silicone matrices being increasingly investigated due to their excellent biocompatibility and low skin irritation potential. A quite broad selection of commercially available products can be found within all three chemical groups, but detailed information on the polymer structure and the excipients present in the material are either insufficient or undisclosed. Limited research focusing on the correlation between the polymer structure and its interaction with APIs is available, which, even in the case of controlled release formulations, makes the primary selection of the optimal polymer based on a lucky guess rather than on an evidence-based methodology.

Currently, the most popular are transdermal patches wherein the API is incorporated in the adhesive matrix, since the coating of the non-adhesive matrix with an adhesive layer involves a more complex technology [1]. API may be present in the polymeric matrix in a crystalline or amorphous form, or it can be dissolved. The dissolved fraction is critical to enable the diffusion of the API onto the skin surface from where it can be absorbed [5]. The high release and absorption rate may be retained due to the saturation of the matrix, which occurs in the presence of the coexisting suspended and undissolved fraction of the API, because the solute flux can be proportionally enhanced by increasing the thermodynamic activity of the drug in the vehicle according to the Higuchi equation [6]. The prediction of an API solubility in a polymer without experimental work is very difficult or impossible, especially taking into consideration the diversity of the polymer chain constructions, even within a single chemical group [7,8].

Considering the above, the evaluation of transdermal patches should consist of an investigation into the APIs’ capability to dissolve in the matrix, as well as towards the determination of the particle size and distribution of the non-dissolved fraction, combined with detailed observations of drug–polymer interactions [9]. Among others, microscopic techniques were reported to be used in order to estimate the solubility of APIs [10,11,12,13]. The observation of the physical state of an API in the patch should enable determining the changes which might occur during storage or during its relatively long (up to several days) application on the skin. Additionally, tools for confirming the identity of the API in medicinal products are especially valuable nowadays, when incidents of counterfeit products are becoming more common.

This paper describes a comparative analysis of PSA polymers, namely silicone and acrylate matrices, intended for transdermal patch formulations. A number of spectroscopic and microscopic techniques were selected to visualize the effect of the matrix type on the fraction of the dissolved API. Previous works have been mostly limited to the evaluation of one type of polymer with a selected active substance, while a more comprehensive approach was hardly ever presented. For this work, two silicones and three polyacrylates were chosen due to their versatile physicochemical properties (the differences in the structure and properties described in Table 1). Three APIs of different lipophilicity and potential for transdermal delivery were investigated (Table 2).

The following microscopic techniques were employed: optical microscopy, scanning electron microscopy and microscopy with a Raman spectrometer. The microscopic observations were supported by DSC studies performed in order to determine the solubility of the API and the potential interactions of the drug with the polymer. In the choice of the matrix-forming polymer, the possibility of a simple identification technique of the polymer type and active substance as part of the product control must also be considered. The spectral evaluation of the selected PSA matrices, with or without an API, was carried out to demonstrate the possibility of using the FTIR and Raman spectrometry for the identification of medicated patches.

## 2. Materials and Methods

### 2.1. Materials

The materials used for patch formulation were as follows. DuroTak^®^ 387-2287 (A1), 87-4098 (A2), 87-2852 (A3) acrylic adhesives solutions were gifted by the manufacturer (Henkel, Brussels, Belgium). Standard Silicone Adhesive solution Bio-PSA MD7-4502 (S1) and two-part (A&B) silicone elastomer Liveo^TM^ Soft Skin Adhesive MG 7-9850 SSA (S2) were provided gratis by DuPont (Brussels, Belgium). Polyethylene (PE) membrane (Esselte, Warsaw, Poland) was used as a backing layer and fluoropolymer-coated Scotchpak^®^ 1020 as a release liner (3M, Neuss, Germany). APIs were: indomethacin IND (Sigma–Aldrich, Steinheim, Germany); testosterone TST (Ipca Laboratories, Mumbai, India); and cytisine CYT (Xieli Pharmaceutical, Sichuan, China). Characteristics of the polymers used for the adhesive matrices are presented in Table 1 and APIs are characterized in Table 2.

### 2.2. Preparation of the Patches

IND, CYT or TST were added to the adhesive polymer solutions A1, A2, A3 and S1 in a concentration of 5% (*w*/*w*) based on the polymer dry weight. Each blend was then mixed in a planetary mixer (ARE-250 Thinky, Tokyo, Japan) for 4 min at 2000 rpm and defoamed for 2 min at 2200 rpm, before being transferred to high vacuum for 5 min to remove the remaining air bubbles. In the case of the S2 matrix, part A was preliminarily mixed with the APIs at 500 rpm for 2 min, and then part B containing a crosslinker was added, mixed and de-aired as described above. The blends were then casted on the PE membranes using a lab coater (Camag, Berlin, Germany) with the gap adjusted to obtain a dry patch thickness of approximately 150 µm. The step of solvent evaporation for A1, A2, A3 and S1 films was carried out in a drying oven at 40 °C for 3 h and then for 12 h at 23 ± 1 °C. The curing process (cross-linking of S2) was conducted at 23 ± 1 °C for 12 h in a high vacuum. The prepared films were covered with a release liner, packed into polyethylene bags with a tight zipper closure, stored in a controlled environment (25 ± 1 °C, RH 60%) and analyzed within 7–30 days. The thickness of the obtained films was accurately measured with an infrared gauge MiniTest 730 (Electro Physik, Cologne, Germany).

### 2.3. Raman Spectroscopy and Confocal Raman Microscopy

A WITec Alfa300 Access microscope with Raman spectrometer (WITec, Ulm, Germany) equipped with a 785 nm laser was used to acquire spectra of the polymeric adhesive patches and APIs. Mainly Zeiss objectives (×10, ×50, ×100) were used for the measurements, with a laser power of 60–80 mW. Data analysis was performed using WITec Project Plus software.

Raman spectra of the drug-loaded acrylate and siloxane polymeric films were collected and compared with the spectra of reference APIs and pure polymer films, taking into account non-overlapping peaks.

A Raman imaging mode was employed to visualize the API distribution in polymeric matrices. Zeiss 50×/NA 0.75 was used for Raman spectroscopy acquisition. The laser was focused on the adhesive patch surface to avoid backing layer interference. In each sample, three randomly chosen 50 µm × 50 µm areas were scanned with a 1 µm step size with an exposure time of 1 s. The Raman maps were processed to reduce non-chemical effects.

For a given API-loaded patch, spectra were normalized to the maximal intensity of the API’s characteristic/signature peak. To generate intensity maps, the signature peaks for each API were selected as non-overlapping with the peaks of the polymer matrix, and a center point of a 10 cm^−1^ range was used. For CYT, a signature peak at 1210 cm^−1^ was chosen for all investigated polymers. In the case of IND, 745 cm^−1^ was found to be suitable for polyacrylates, while the 1700 cm^−1^ band allowed to identify API in silicone polymers. The TST spectra showed the 950 cm^−1^ peak as a signature in all polymers except for A2, where only the 1615 cm^−1^ peak did not interfere with the polymer spectrum.

### 2.4. Scanning Electron Microscopy (SEM) and Optical Microscopy

To assess the uniformity of particle distribution, crystallization and morphology of the particles, the microscopic images of the films surface were obtained using an optical microscope (Nikon Eclipse i50, Nikon Instruments, Tokyo, Japan). Zeiss objectives 10× were used for a general overview of the sample and the images within an in-depth range of 15 μm were obtained with Ph2 DLL 40× magnification lens. The images that were captured in a different axial (z) dimension were processed with NIS Elements Advanced Research 3.20 software (Nikon Instruments, Tokyo, Japan).

Details of the patches surface were observed with a scanning electron microscope (Phenom Pro Generation 5, Thermo Fisher, Eindhoven, The Netherlands) using an in-line detection mode at 5–10 kV, with a backscattered (BSD) or secondary electron detector (SED). Samples of approximately 5 × 5 mm were coated with a thin layer of gold in an ion-sputtering device (thickness 5 or 10 nm).

### 2.5. Fourier Transform Infrared Spectroscopy (FTIR)

FTIR spectra were obtained using Jasco-4700 instrument (4000–400 cm^−1^ with 32 scans, 4 cm^−1^ resolution; Jasco Company, Tokyo, Japan). Thin film method using salt (KBr) plates was applied by dissolving 10–20 mg of sample in 1–2 drops of solvent mixture (ethyl acetate 65%, isopropanol 19%, hexane 12%, toluene 3% and benzene 1% *v*/*v*) and placing one drop of the solution on one salt plate. After evaporation, the analysis was performed. The spectrum from a clean plate was recorded as a background and the analysis of spectra was performed using Spectra Analysis software (Jasco Company, Tokyo, Japan).

### 2.6. Differential Scanning Calorimetry (DSC)

DSC thermograms were obtained using the DSC-1 STARe System (Mettler Toledo AG, Schwerzenbach, Switzerland) combined with the intercooler system (HUBER TC 100) and the program STARe Evaluation Software version 16.30. All patches samples (10–13 mg) or pure APIs (0.5 mg) were sealed in flat-bottomed aluminum pans (40 µL). The indium calibration standard was used to calibrate the DSC instrument. A 75 mL/min nitrogen flow was used and the heating rate was 5°C/min. The analytical temperature range was from −40 °C to +350 °C. The thermogram recorded for an empty pan was treated as a baseline. The melting temperature (T_m_) and glass transition temperature (T_g_) values were determined as the midpoint of the endotherm and inflection in the DSC thermograms, respectively.

In an additional experiment, thermograms were recorded for a sample prepared by layering 0.5 mg of API next to the placebo acrylate or silicone patch, without mixing. The enthalpy (ΔH) was compared with enthalpy determined for the drug-loaded patches and for pure API (0.5 mg).

## 3. Results

### 3.1. FTIR Spectroscopy

**Figure 1:**
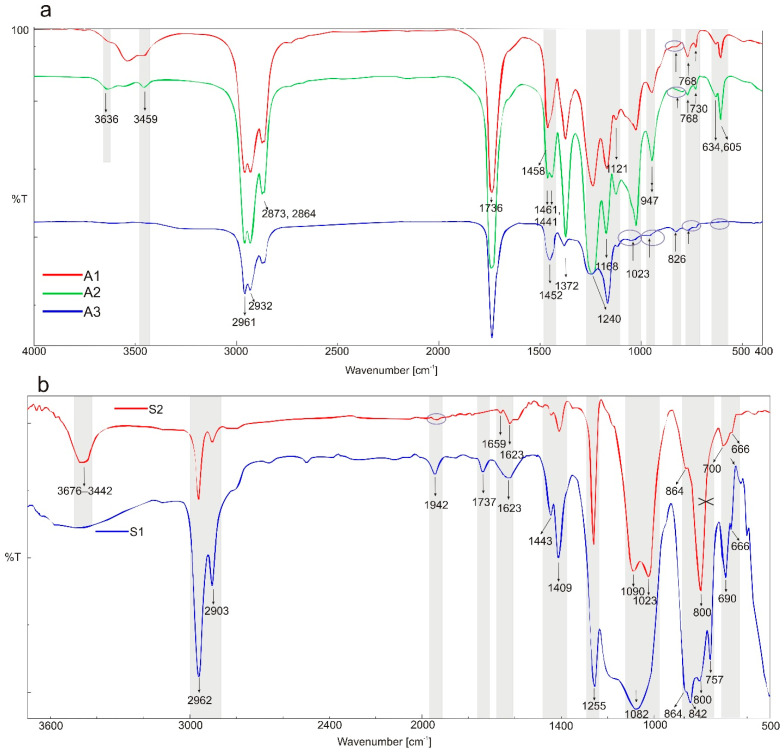
FTIR spectra of the investigated placebo patches: (**a**) polyacrylates; and (**b**) polysiloxanes. The grey bands mark differences or similarities between the polymers’ spectra.

Figure 1 shows the IR spectra of the tested placebo patches: three acrylic polymers and two siloxane polymers [18]. All characteristic bands according to chemical structure of the material were identified and presented in Table 3. Spectra of all tested polyacrylates are characterized by a wide band (wavenumber at 3700–3000 cm^−1^) corresponding to the stretching vibrations of the hydroxyl group (free OH group from water and hydrogen bonds). In this region, both acrylic or silicone matrices present typical FTIR spectra bands between 3000 and 2800 cm^−1^, corresponding to asymmetric C-H (CH_3_); specific ranges for the carbonyl acrylic group C=O are observed at 1736 cm^−1^; while vinyl group from silicone derivative structure CH_2_ is visible at wavenumber 1409 cm^−1^. Characteristic −CH_2_, −CH_3_ and −CH deformation vibrations (1450–1372 cm^−1^) and ester group vibrations O-C for acrylate polymers are observed at 1121 and 1168 cm^−1^. Silicone matrices present typical FTIR spectra corresponding to a C-H band for Si-CH_3_ at 1255 cm^−1^, Si-O-Si band at 1092 and 1023 cm^−1^, Si-C from Si-CH_3_ at 864 and C-H (CH_3_) at 800 cm^−1^. Other bands are not specific and their presence results from a solvent or other additives.

**Figure 2:**
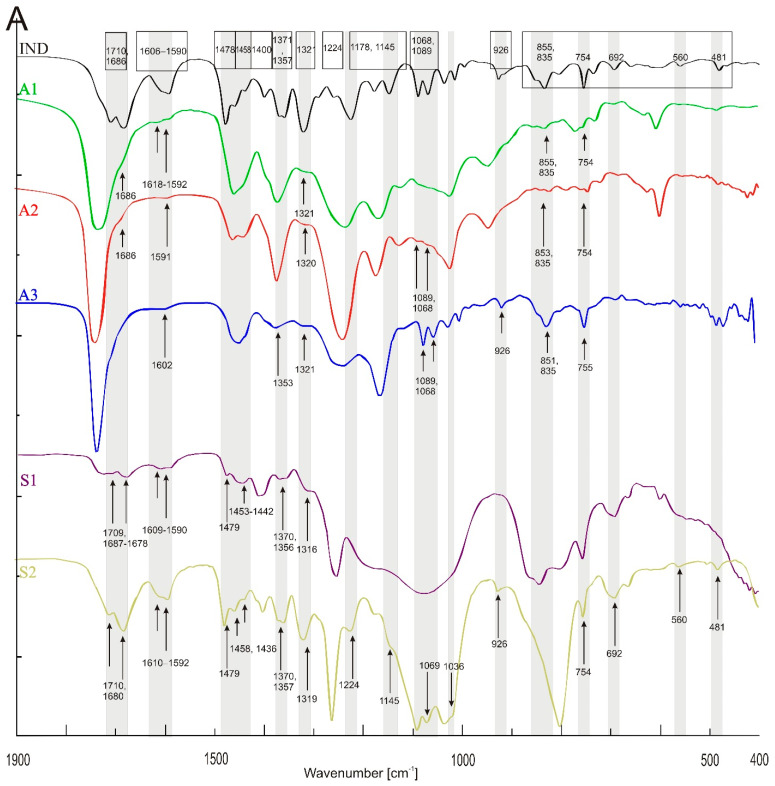

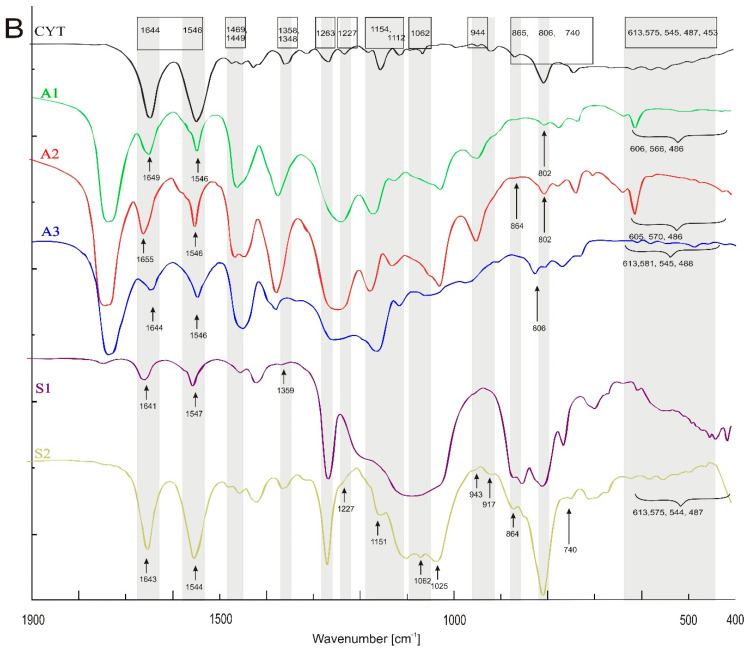

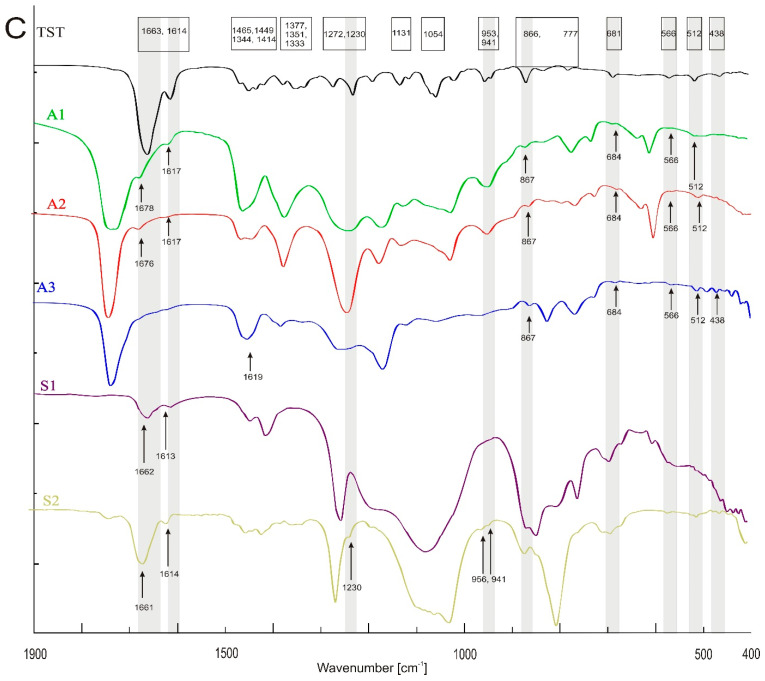
FTIR spectra of polymeric patches with API: (**A**) indomethacin, (**B**) cytisine, (**C**) testosterone.

Infrared spectra of the tested patches containing 5% *w*/*w* of API are presented in Figure 2A–C. Each spectra of placebo polymeric matrices (acrylic A1–A3) compared to patches with APIs spectra are collected and presented in Supplementary Materials Figures S1–S5. In Table 3, all bands identified in the patches loaded with three different APIs are listed. Generally, all investigated polyacrylic patches allow one to observe the presence of the incorporated API. In the spectra of IND patches (Figure 2A), the following bands were characteristic for API: near 1618–1591 cm^−1^ (C=O benzoilo amide), at 1321 cm^−1^ corresponding to C-N vibrations, at 1089, 1068 and 835 cm^−1^ for C-Cl bond, and 754 cm^−1^ for C-H bending. For CYT in the patch, only three characteristic bands were observed in the spectra (Figure 2B), namely N=C=O at 1644 cm^−1^, cyclic -C=C- at 1546 cm^−1^ as well as in wavenumber the range of 806–802 cm^−1^. TST can be identified in acrylic matrices only based on the characteristic -C=C- cyclic alkene bands at wavenumbers: 1619–1617, 867, 684 and 566 cm^−1^ (Figure 2C).

### 3.2. Raman Spectroscopy

**Figure 3:**
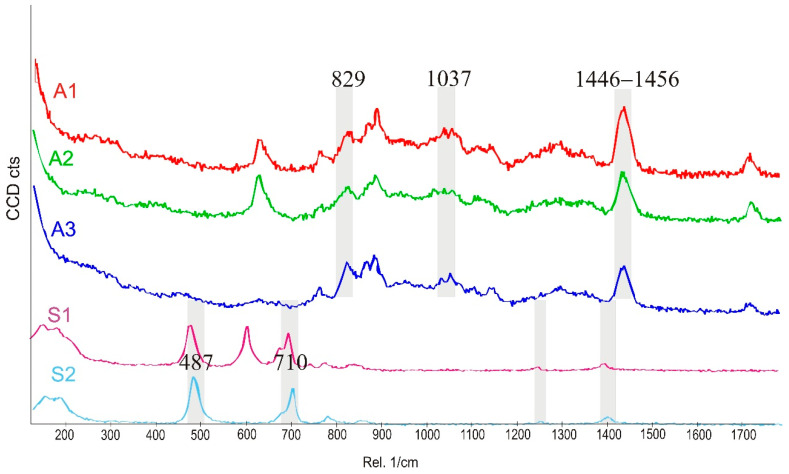
Raman spectra of placebo patches: polyacrylates (A1–A3) and polysiloxanes (S1, S2). The grey bands mark differences or similarities between the spectra of the polymers.

The Raman spectra are complementary to the FTIR spectra described above [23]. In Figure 3, spectra of placebo polymer matrices are presented. Generally, the stretching vibrations C=O at 1740 cm^−1^, −CH_2_, −CH_3_ and −CH deformation vibrations at 1446–1456 cm^−1^ as well as at 830, 633 (A2), 526 (A1) are observed in acrylates. Vibration of “additional” CH_2_ group corresponds to the band near 633 cm^−1^ for A2 and S2 at 614 cm^−1^ observed also in the FTIR spectra.

The data compiled in Figure 3 and Table 4 show that the spectra of polysiloxanes are less complex and have more potential space for the observation of the incorporated APIs. Practically, for all three APIs, all of the most characteristic bands are visible in the Raman spectra. Moreover, in the case of IND, an additional signal at 1698 cm^−1^ (characteristic for a benzoilo −C=O)—absent in polyacrylates—was identified. Notably, the Raman band at 487 cm^−1^ in S1 and S2 is derived from nanosilica particles embedded in the silicone matrix.

The characteristic bands of the investigated APIs in Raman spectra are also listed in Table 4a–c, together with the bands identified in the API-loaded patches (the spectra are presented as Supplementary Materials—Figure S6). In all acrylic patches, the IND signals were identified at 734–737 cm^−1^ for C-H bending. In the spectra of IND in A3 and A2, additional specific bands at 1358–1361 cm^−1^ (O-H group) and at 1220–1223 cm^−1^ corresponding to C-O-C vibrations were shown. For CYT in acrylates, there is -C-O-C stretching vinyl ether or −C-N- at 1207–1222 cm^−1^ and −C=C- near 613 and 659 cm^−1^. TST is observed in the acrylic matrices due to its C-H- methyl group at 1611 cm^−1^. This is the only signal of TST in A3. Additional signals in A1 and A2 with TST appeared near 1663 and 1000, 946 cm^−1^ corresponding to −C=C- cyclic alkene and C=C bending, respectively.

### 3.3. Optical Microscopy, the SEM and Raman Mapping

**Figure 4:**
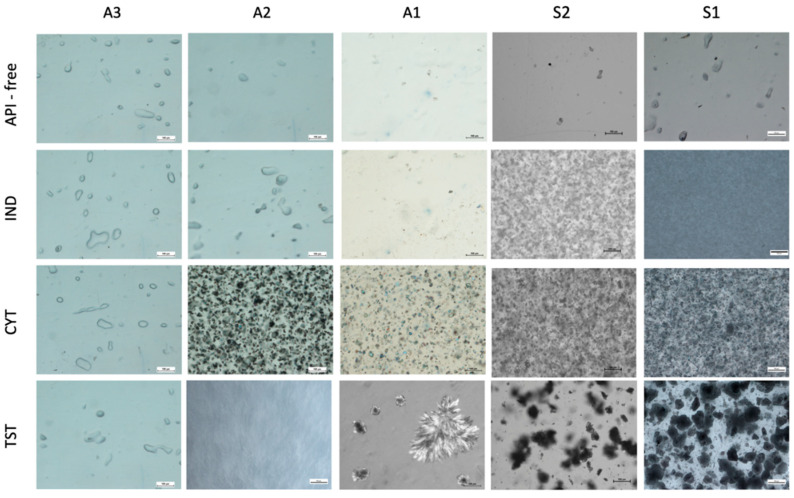
Optical microscope images of acrylate and silicone patches (scale 100 µm).

The microstructure of all five polymers mixed with three API was investigated by optical microscopy and compared with the API-free films, as shown in Figure 4. Each optical image was taken within 15 μm depth of focus, starting at the sample surface. The API-free films were transparent, and free from solid particles. Due to the roughness of the patch surface, the backscattered light effect could be observed as irregular spots on the optical microscope images. Acrylate patches demonstrated a much rougher surface, with multiple parallelly orientated “furrows”, while silicones were smooth, as visible under a SEM.

In the silicone matrices, the shape and size (below 10 μm) of the API particles did not change when compared with the particles of the raw material used for the preparation of the patches. It was only in the case of silicone patches with TST that the agglomerates were observed.

By contrast, the optical images of polyacrylic polymers showed diverse interactions, mainly dependent on the type of API. Only IND patches prepared with all three polyacrylates (A1–A3) were transparent, without visible particles. The material of patch A3 was the one which also enabled the dissolution of TST and CYT producing clear patches. In the case of CYT the patches, A1 and A2 showed the homogenous distribution of solid particles; however, the number of particles in CYT-A1 images appeared to be significantly smaller. No re-crystallization of CYT was observed. In contrast, during the first 24 h of storage, the TST re-crystallized in A1 and A2 patches: needle-shaped crystals were homogenously distributed in the A2 patch, while in A1, it formed large crystal aggregates.

**Figure 5:**
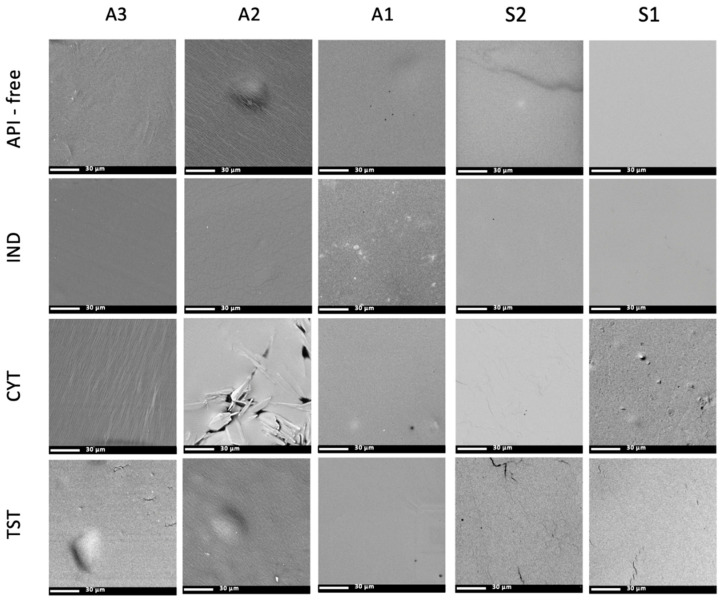
SEM images of acrylate and silicone patches (scale 30 µm).

A comparative analysis of the SEM micrographs with optical images allowed to obtain additional information on the location of the crystalline API. Only in the case of A2 with CYT, a considerable number of drug particles was located superficially, whereas the TST crystals present in A1 and A2 were not observed using the SEM (Figure 5) [24].

The detailed study of the microstructure of the adhesive patches continued using a SEM, where the area of interest was the patch surface. As shown in Figure 5, the microstructure of a polymer patch in the presence of an API undergoes subtle but noticeable changes. Acrylate patches presented a much rougher surface, with multiple parallelly arranged “furrows”, while silicones were smooth. On the other hand, pin-hole-like structures were only present in the patches prepared by solvent evaporation and were not spotted in the S2 matrix.

**Figure 6:**
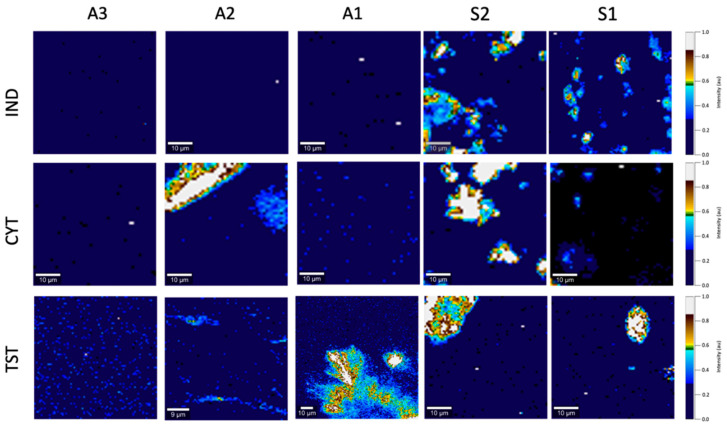
Raman image of indomethacin (IND), cytisine (CYT) and testosterone (TST) distribution on the surface of acrylate (A) and silicone (S) patches (scale 10 µm).

Detailed Raman maps were generated (Figure 6), where the intensity of the Raman signatures of IND, CYT or TST enabled the location of the regions with the API. In probing with a confocal system of a depth-of-focus of ca. 1–2 µm, an image of the thin surface layer of the patch was created.

In the case of polyacrylates, IND was found to be present on the patch surface forming a homogenously distributed layer of low intensity; a similar image was recorded for A3/CYT. Single particles, their diameters below 1 µm, unnoticed under the imaging techniques described above (SEM and optical microscopy), were occasionally identified. The CYT distribution demonstrated the most pronounced variation. CYT in the A2 resembled elevated particles observed under the previous imaging techniques, while for A1/CYT, the particles were found near the surface, probably still covered by the polymer, as proven by plain SEM micrographs (Figure 5). A noticeable decrease in the TST concentration in the polymeric matrix was found in the case of A1 and A2, which was due to crystal formation in the deeper patch layers. Especially in the A2, some regions devoid of TST presence in the surface layer were identified. Surprisingly, in the A3 patches, a considerable number of evenly distributed TST-rich areas were noted, which indicated the presence of TST particles sized below 1 µm, undetected by the other imaging techniques used.

Silicone polymers exhibited the most noticeable fluctuations of the spectra: alongside stronger signals, from the sites corresponding to the API particles, a gradual drop in signal intensity was recorded in the areas surrounding the particles (the ‘hallo’ effect). Due to the dominating fraction of solid drug particles in the S1 and S2 silicone patches, numerous black spots representing drug-free areas were observed in the distribution maps (Figure 6). In the case of S1/CYT, an undetectable amount of dissolved API was observed superficially, with only single particles submerged in the polymeric matrix.

### 3.4. DSC

**Figure 7:**
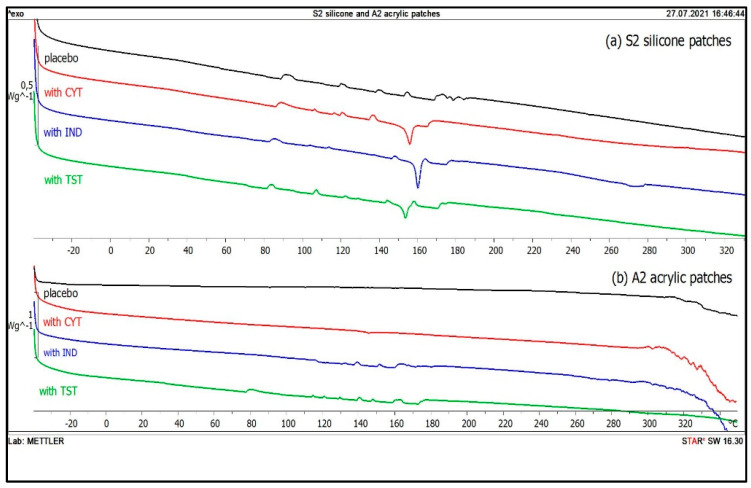
DSC thermograms of S2 silicone patches (**a**) and A2 acrylic patches (**b**): placebo (black), with CYT (red), IND (blue), TST (green).

Clear endotherms for all investigated pure APIs were observed. The determined melting temperatures, T_m_ (Table 5), corresponded to the values reported in the literature [25]. Thermograms were also recorded for drug-loaded silicone patches (S1, S2) and endothermic peaks with T_m_ corresponding to T_m_ of pure API were obtained confirming the presence of IND, TST and CYT in the matrices. Sample thermograms for the S2 silicone patches are presented in Figure 7a, where a lack of any significant shift of the peaks compared to pure API indicated the absence of interaction between the incorporated API and the silicone polymers (Table 5).

Contrary to the above, melting endotherms associated with the investigated APIs were absent in the acrylate patches (Figure 7b and Table 5). The effect might have been predicted in the case of a matrix where the API was dissolved and microscopic observations did not reveal any visible particles (all A3 patches, and IND in A1 and A2 patches). Interestingly, although solid API particles were present in the acrylic patches with CYT and TST (Figure 4), endotherms were missing in their recorded DSC thermograms. In order to explain the reason of the low endotherm intensity of the API in silicone matrices and the lack of endotherm noted for acrylate patches, further investigation was performed, whereby 10 mg of API-free polymer was placed in aluminum pans with 0.5 mg of CYT (to simulate 5% *w*/*w* API content in the patch). The measured enthalpy of CYT (ΔH) was very low (ranging from −4.0 to −5.2 J/g) compared to the value measured for the same amount of pure CYT (−73.2 J/g). Moreover, no differences were observed linked to the presence of a particular polymer. In the Supplementary Materials (Table S1), more detailed results are presented, demonstrating that, by increasing the amount of API (CYT or IND) in the test pan, proportionally higher enthalpy was observed. 

Further investigation consisted in the glass transition (T_g_) determination of the acrylates, as presented in Table 5. Due to equipment-related limitations (operating temperature range: −85 °C–350 °C), the T_g_ of silicone matrices could not be determined (T_g_ below −120 °C [26]). A significant decrease in T_g_ of the A2 and A3 polymers was noted in the presence of all investigated APIs. Interestingly, the T_g_ of the A1 was found to be much lower compared to A2 and A3; moreover, no significant shift was observed in the presence of the API.

## 4. Discussion

Despite the fact that adhesive matrices have been used as a drug carrier in the transdermal delivery of APIs for almost four decades, surprisingly limited research has been dedicated to evaluating the advanced physicochemical parameters of those polymeric materials when combined with the API.

Various physicochemical properties of either the polymer or the API may influence the structure or appearance of the patches. At some technological stages, such as the evaporation of the solvent during the thin layer formation, uncontrolled re-crystallization may occur. Some effects may also result in changes in the microstructure of the polymer matrix. Three complementary methods: optical microscopy, scanning electron microscopy and Raman mapping were employed to study the appearance of adhesive patches with the emphasis laid on the physical state of the API and its distribution in the matrix.

The matrix PSA materials under investigation differed not only in the chemical structure, but also in the process of forming the adhesive films. The investigated acrylic PSA polymers (A1–A3) contained organic solvents (Table 1), which were evaporated in order to form an adhesive patch. Similarly, one of the silicone PSAs (S1) contained a solvent, while another one (S2) formed a matrix in a cross-linking reaction between its two components. It was expected that these differences might influence both the microscopic organization of the polymeric matrix and the solid state of the API in the final patch formulation.

When the patches were observed under optical microscopy, it was found that silicone patches were smooth and acrylate patches presented a much rougher surface. Such differences between the acrylic and silicone matrices may result from the different plasticity of the materials and the microdeformation of the surface of the acrylate patches occurring during solvent evaporation in the course of the preparation process. Acrylate may be more susceptible to stretching and shrinking due to polymer chain mobility. On the other hand, pin-hole-like structures were only present in the patches prepared by solvent evaporation.

### 4.1. Silicone Patches

In regard to the data obtained with the imaging techniques, all model APIs, independent of their lipophilicity or the silicone matrix type, were present in the S1 and S2 as suspended particles. The SEM revealed the location of the particles in the inner part of the polymeric matrix. From the shape and size of the particles, it was concluded that neither significant dissolution, nor other interference from the silicone matrix occurred. This also indicates that in the silicone patches, the dissolved fraction of the API capable of diffusing was inconsiderable. The residue solvent in the S1 was considered irrelevant, since neither API dissolution nor crystallization occurred, even though the latter effect has been suggested by other authors [27]. The distribution of the particles was noticeably homogenous with sufficient incorporation into the matrix, a fact confirmed by SEM, since no particles in the images of the patch surface were noted, even for TST, where the formation of agglomerates had been confirmed in optical micrographs. The mentioned agglomerates pose a problem in terms of the product quality and indicates the necessity of adding a levigating agent or a surfactant to the TST formulations, a step not needed for less lipophilic APIs.

Silicone matrices give IR and Raman spectra containing relatively few bands since the functional groups in this type of polymer are rather predictable. This makes it possible to easily identify the active substances present in the patch. It is noteworthy that, in contrast to the Raman spectroscopy, a direct FTIR analysis of APIs in the adhesive patches proved unfeasible. Hence, a method of preparing samples was proposed: before recording the spectra, the patch samples were dissolved in a mixture of organic solvents, the solution was placed on a salt plate, and a FTIR analysis was performed after evaporation.

Numerous bands were identified in the drug-loaded patches spectra that can be used for routine patch quality control. This is especially the case for S2, where almost all bands specific for the three APIs are visible (Figure 2A–C and Figure 3 and Figure 4). For the S1 and S2 matrices, the wavenumber region between 1900 and 1300 cm^−1^ is the most suitable for analyzing the added substances. In this region, the IND bands at 1709 cm^−1^ (characteristic for the symmetric benzoilo amide −C=O group) and at 1478 cm^−1^ (the olefin group -C=C-) are observed. An interesting result of a comparison between all polymeric matrices is noticed for the −C-O-C- group of IND, because only S1 allows to observe it at 1224 cm^−1^. Inversely, it does not allow seeing the −C-Cl- at 1068/1089 cm^−1^. For CYT, the most characteristic bands of the N-C=O and −C=C- cyclic alkene group vibration are visible at 1644 and 1546 cm^−1^, and CYT lends itself to much clearer identification in the S2, then in S1, as the following bands are sharply visible in the FTIR spectrum: at 1644 cm^−1^ (amine), 1546 (pyridone −C=O), 1166 (C-N), 1151 (C-N stretching amine), 943, as well as a group of bands: 613, 575, 544, 487 (−C=C- aromatic), and 806 (amine). On the other hand, TST is easily observed only at 1663 cm^−1^ (C=O stretching vinyl/phenyl ether) and 1617 cm^−1^ (−C=C- cyclic alkene) in both polysiloxanes patches, while other bands (from −C-O-C- or -C-O at 1230, 953 and 941 cm^−1^) are hidden in the S1 but strongly manifested in S2.

Similarly to the FTIR analysis, in the Raman spectra, practically all characteristic bands are visible for all three APIs in the silicone matrices.

Raman mapping is considered especially useful for identifying APIs and excipients in solid state mixtures [28]. In this study, the patches were analyzed not only for the homogeneous distribution of the undissolved particles, but also with the aim of identifying regions with dissolved API. For example, the absence of CYT signals apart from the particles identified in the S1, with no such effect in the S2, would indicate that both silicone matrices differed significantly in terms of solubility of this hydrophilic drug substance. Silicone polymers exhibited noticeable fluctuations of the API spectra in microscopic images: in addition to stronger signals from the sites corresponding to the API particles, a gradual drop in intensity in the areas surrounding the particles was recorded. Such a ’hallo’ effect can result from lower API concentration and may be attributed to the saturation of the matrix with dissolved API at the particle/matrix interphase; however, it could also represent the ’iceberg’ effect.

The use of silicone matrices in which drug substances form a suspension also allows their easy identification using the DSC method. The endotherms of all three APIs in the silicone patches were visible on the thermograms and the lack of shifts signaled that there was no API interaction with the silicone matrix during the preparation of the patches. Moreover, the lack of marked T_g_ for the API also indicates the absence of amorphization due to the contact of the API with the matrix components.

### 4.2. Acrylic Patches

In contrast to the silicone matrices, greater differences were observed in acrylate matrices, both as a result of the differences in the properties of the active substances, and in the varieties of the polymer materials used. It can be concluded that, especially in the case of these materials, it is difficult to predict what physical system will be created as an effect of introducing the active substance. It was only in the case of IND that a solution system was formed in each of the three investigated matrices. It was demonstrated by the DSC that in the case of the A2 and A3 matrices, the dissolved IND plasticized the matrix reducing the glass transition temperature, which is evidence of the API interacting with the matrix polymer.

In the case of the acrylic patches, where an API is dissolved in the matrix, the applicability of the microscopic techniques is limited to the evaluation of the re-crystallization of the API, as was noted for TST in the A1 (Figure 4). Although the only structural variation claimed for the A1 polyacrylate is the presence of the hydroxyl group in the polymer chain, it was considered insufficient to explain the stable dissolved state of the TST in the case of the A2 and A3 matrices. Since other additives are present in the polymer solutions, undisclosed by the manufacturer in terms of their quantity or type, those variations in composition may partially influence the solubility of the API in the final matrix. Optical microscopy allowed for not only detecting the undissolved TST particles, but also precisely demonstrating the shape of the crystals, which indicated the re-crystallization of the originally dissolved TST. Interestingly, a further detailed comparative analysis of the SEM micrographs with optical images allowed obtaining additional data on the location of the crystalline API. The examination of the patch surface using an SEM excluded the presence of the TST (Figure 5), which suggested the location of the crystals in the deeper layers of the matrix and proves that the re-crystallization of TST occurred not on the air/patch contact surface but in deeper layers of the polymeric matrix. The observation leads to the conclusion that re-crystallization was not initiated by external conditions (e.g., contact with air or mechanical stress) as previously observed for fentanyl patches [24]. One cannot rule out that the observed phenomena for TST in the acrylate patches indicate that the dissolved fraction is present in the concentration close to the saturated state, which should result in high thermodynamic activity and thus an optimal release rate [13].

The differences in the potential capability of the acrylate polymers to dissolve the APIs were most profoundly shown for CYT (Figure 4, Figure 5 and Figure 6). All microscopic images showed that CYT was undissolved in the A2 and A1 matrices, but the number of particles in the CYT-A1 images appeared to be significantly smaller. This suggests the increasing solubility of CYT in polyacrylic matrices in the following order: A2 (undissolved) < A1 (partially dissolved) < A3 (dissolved). The complete dissolution of CYT was confirmed only in the case of the A3 matrix, which differs from A1 and A2 not only due to the presence of the COOH functional group in the polymeric structure, but also in that it contains a mixture of solvents (Table 1). CYT is the most hydrophilic substance among the examined APIs (Table 2) and in this case, the choice of proper polymeric material for the acrylic patch formulation is crucial for the development of a product with the API in an expected physical state.

The Raman maps provided some additional information on the presence of the API particles of the diameter below 1 µm, unnoticed by the SEM or optical microscopy. Such particles were occasionally identified even in the patches with IND which, on the basis of other microscopic techniques, were considered to contain the API in the dissolved state.

Compared to silicones, the acrylic polymers contain more functional groups, and the placebo patches give IR and Raman spectra with numerous specific bands, which makes the identification of the API in the patch by spectral methods more difficult. Nevertheless, FTIR and Raman spectra were obtained for each of the active substances tested, enabling API identification. The spectral analysis also distinguished three placebo matrices. In this case, the samples obtained after dissolving the patch and evaporating the solvent were also needed to record the FTIR spectra.

In the case of IND, the characteristic bands identified in the silicone patches at 1709 cm^−1^ and at 1478 cm^−1^ were absent for polyacrylates. Notably, the band near 1686 cm^−1^ was absent in the A3 matrix. Acrylic polymers also hide the vibration of IND’s O-H bending alcohol due to its own vibration in this area of the spectrum. For CYT, the vibration of the most characteristic bands of the N-C=O and −C=C- cyclic alkene group was visible at 1644 and 1546 cm^−1^, both in silicones and acrylates; however, the band at 1360 cm^−1^ (−C-N- stretching) present in the S1 silicone patch was fully covered in the acrylic patches. Similarly to the silicone patches, the TST band is easily observed at 1663 cm^−1^. Like in the FTIR analysis, all polyacrylic matrices show the presence of added APIs in the Raman spectra.

The data obtained using the DSC indicate a vast difference in the state in which API occurs in silicone and acrylic polymers. Despite the same low API content in the investigated matrices (5% *w*/*w*), crystalline form was confirmed only in silicone matrices. Even if the particles of suspended API were present, as in polymer A2 with CYT, or when the re-crystallization of TST in the A1 and A2 occurred, no API endotherm was noted. The lack of API peaks in the thermograms of the acrylic patches leads to the conclusion that a substantial amount of the API was dissolved in the acrylic matrices and the signals from undissolved particles were too weak.

It may also be concluded that due to the insufficient sensitivity of the DSC method and the excessively low API concentration, the particles visualized by microscopic techniques in the A1 and A2 were not identified in the form of endotherms. In similar cases of a decrease in peak intensity and enthalpy, only few researchers to date have addressed the issue and the suggested relevance of low API content [29], the loss of API crystallinity due to its incorporation in dissolved form [30], API dissolution in the matrix during the melting in the DSC analysis [31], or phase transition (e.g., amorphization) resulting from interaction with the polymer [32]. However, considering the above, the amorphization of the API was neglected in the case of all matrices, since no glass transition, T_g_ was noted on the thermograms for the investigated APIs.

In spite of the failure to identify the API in acrylic patches, the DSC technique appeared to be useful to observe the interaction between the API and the polymer. The effect of API on the physical state of the acrylic matrix was demonstrated by the changes in T_g_ of the polymers. A significant decrease in T_g_ in the A2 and A3 polymers was noted in the presence of all investigated APIs, which indicates the increased polymer chain mobility caused by the API plasticization effect [33,34,35]. Moreover, it was assumed that the lowest T_g_ of the A1 polymer (T_g_ = −26 °C) initially indicates its most elastic properties among the analyzed viscoelastic materials, and in this case, none of the APIs could further increase mobility of the polymer chain.

## 5. Conclusions

The present study indicates that the applied techniques are valuable for identifying APIs in transdermal adhesive patches, and potentially useful for the quality control of the medicated patches.

The modern microscopic and spectroscopic techniques enable the broad characterization of the adhesive matrices and the observation of the active substance distribution within their structure. Among the employed spectroscopic techniques, FTIR and Raman may be used not only as standard methods for API identification in the matrix, but also to distinguish commercially available polymeric materials of a similar chemical structure. The spectra of the silicone and polyacrylate polymers presented in this work may be especially useful as a reference whenever the information provided by the manufacturers of the materials is insufficient. The presented methodology is considered as an essential tool for the precise determination of the polymer identity. Moreover, the proposed procedure for the sample preparation for FTIR analysis was developed and optimized. The results of the performed spectroscopic analysis prove that it is useful to introduce the IR and Raman methods in the routine quality control of the patches because of the simplicity of the identification they offer and their uncomplicated methodology.

Although the evaluation of the API state in the matrix or the distribution, morphology and particle size of the undissolved fraction can be defined by optical microscopy, the surface-focused SEM can provide additional information on the three-dimensional location of the API, which can be helpful, e.g., in interpreting the release kinetics. As a single microscopic technique, the Raman microscopy not only allows producing images of the undissolved particles, but is especially valuable in the evaluation of the dissolved fraction distribution within the matrix.

The DSC was the only tested technique which failed to identify the API in the acrylic patches, even if undissolved API was present. However, in the case of silicone patches, where the API endotherms were recorded, it enabled the confirmation that the API was practically undissolved, while measurements of the T_g_ values of the acrylic matrix polymers indicated that dissolved API could act as a plasticizer in some types of matrices.

## Figures and Tables

**Table 1:** Characteristics of the commercial polymers used for the preparation of adhesive matrices [14,15,16].

PSA Type	Acrylate			Silicone	
Symbol	A1	A2	A3	S1	S2
Brand name	DuroTak^®^ 387-2287	DuroTak^®^ 87-4098	DuroTak^®^ 87-2852	Bio-PSA MD7-4502	Soft Skin Adhesive MG 7-9850
Film formation	Solvent evaporation				Polymerization
Structure and chemical name	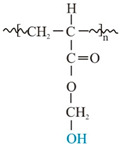 Acrylate-vinylacetate	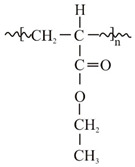 Acrylate-vinylacetate	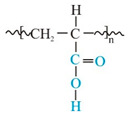 Acrylic acid	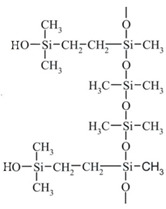	
Additives	Vinyl acetate	Vinyl acetate	Crosslinker	No data	Crosslinker
Solvent	Ethyl acetate	Ethyl acetate	Ethyl acetate, n-heptan, isopropanol, toluen, acetylacetone	Ethyl acetate	--
Content of solids (%)	50.5	38.5	33.5	60–65	100
Viscosity * (mPa*s)	18,000	6500	2500	1500	2900 **

* Viscosity of a polymer solution. ** Viscosity of part A and part B before the curing process.

**Table 2:** Characteristics of the investigated drug substances (APIs) [17].

	Indomethacin (IND)	Cytisine (CYT)	Testosterone (TST)
Chemical formula	C_19_H_16_ClNO_4_	C_11_H_14_N_2O_	C_19_H_28_O_2_
log P/clog P	3.4–4.25	0.6–1.06	2.99–3.37
Molecular weight (g/mol)	357.8	190.2	288.4
Melting point T_m_ (°C)	158–162	152–153	155
Polymorphism	α, β, γ, δ, new forms: ε, ζ, η	No data	4 polymorphic forms
Solubility in water (mg/mL)	0.0024 (pH dependent)	8.14	0.0333
Particle size * (µm)	3.1 ± 2.6	6.6 ± 3.9	5.8 ± 2.6

*—average size (±sd) in the drug powder used in the experiments (experimental data).

**Table 3:** FTIR identification of IND (a), CYT (b) and TST (c) in the polymeric matrices (bands or wavenumber ranges underlined are characteristic of API identification in respective patches and not present in placebo polymer film) [19,20,21,22].

(**a**)											
Chemical Bond	IND 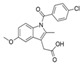	Acrylates						Silicones			
		**A1**	**A1 + API**	**A2**	**A2 + API**	**A3**	**A3 + API**	**S1**	**S1 + API**	**S2**	**S2 + API**
O-H intermolecular bonded	-	3538, 3459	3541, 3454	-	-	3448	3541, 3454	3485	3349	3474	-
C-H (CH_2_) stretching alkane	3088, 2993, 2960, 2933, 2833	2956, 2927, 2875–2859	2956, 2927, 2875–2859	-	-	2956, 2927, 2867	2956, 2927, 2856	2961, 2902	2961, 2902	-	3088
C=O stretching vinylphenyl ester	-	1738	-	-	-	1735	-	1737	1728	-	-
Asymmetric acid −C=O ketone	1709	-	-	-	-	-	-	-	1709	-	1710
(benzoilo)-C=O amide	1685, 1590 1611–1593	-	1686, 1618–1592	-	1686, 1591	-	1602	1644, 1632, 1622	1687–1678, 1609–1590	-	1680, 1610–1592
C-H bending alkane methyl group	-	1462	1459–1414	1463, 1435	-	1465–1448	1454	-	-	-	-
−C=C-	1478, 1458, 1437	-	-	-	-	-	-	1442	1479, 1453–1442	1443	1479, 1458, 1436
C-H (CH_3)_	1400	-	-	-	-	-	-	1414	1413–1401	1409	1411
O-H bending alcohol	1371,1357	1373	1373	1373	-	1380	1378–1353	-	1370, 1356	-	1370, 1357
−C-O- acidic group	1321	-	1321	-	1320	-	1321	-	1316	-	1319
−C-O-C-	1292, 1261, 1224	1238	-	1237	-	1260–1238	1258	1254	1254	1260	1260, 1224
−C-N- or/and C-O stretching ester	1178, 1148	-	-	-	-	-	-	-	-	1180	1175
	-	1166	-	1166	-	1164	1164	-	-	-	1145
O-H alcohol	-	1123	1123	1123	-	1116	116	-	-	-	-
−C- Cl-	1089, 1067	-	1086, 1068	-	1089, 1069	small 1079, broad 1043	1089, 1069	1076	1076	1093	1088, 1069
−C-O-C-	1036, 1015	-	-	-	-	1035	1035, 1015	-	-	-	1036
C-O stretching primary alcohol	-	1023	1023	1023	-	-	-	-	-	1024	1018
C-O vinyl acetate	995	-	-	-	-	-	-	-	-	-	-
	968	944	-	945	945	broad 964	-	-	-	-	-
−C-H-	926, 803, 692	-	-	-	-	-	926	946, 692	926, 692	660	926, 692, 662, 483
strong C-H bending	-	-	835	-	835	-	835	-	-	-	-
−C-Cl- and strong C-H bending	854, 833, 734	-	856, 803	800	853, 800	-	853	864, 842, 803	864, 842, 803	865, 800	866, 846, 800
C-H bending	754, 733	768, 728	755, 728	770	770, 754	772	770, 755	757	757	-	754, 560, 481
(**b**)											
Chemical bond	CYT 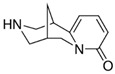	Acrylates						Silicones			
		**A1**	**A1 + API**	**A2**	**A2 + API**	**A3**	**A3 + API**	**S1**	**S1 + API**	**S2**	**S2 + API**
ν (N-H) intermolecular bonded/O-H	3420	3533, 3450	3533, 3449	3628, 3542, 3454	3628, 3542, 3454	3448	3450	3485	3349	3473	3405
C-H (CH_2_) stretching alkane	2944, 2736	2960, 2927, 2875, 2857	2960, 2927, 2875, 2857	2953, 2935, 2879, 2863	2953, 2935, 2879, 2863	2956, 2927, 2867	2956, 2927, 2867, 2736	2961, 2902	2961, 2902	2964, 2902	2964, 2902
C=O stretching vinyl/phenyl ether	-	1737	1737–1726	1745–1725	1745–1725	1737	1737–1726	1736, 1625	-	-	-
N-C=O	1644	-	1649	-	1655	-	1644	1648	1641	-	1643
−C=C- cyclic alkene	1546	-	1546	-	1546	-	1546	1547	1547	-	1544
C-H bending alkane methyl group	1469, 1449, 1425, 1410	1460	1460	1462	1462	1465–1448	1465–1448	1447	1447	1442	1446, 1470
O-H bending alcohol	-	1380	1380	1372	1372	1380	1380	1411	-	-	-
−C-N- stretching	1358, 1348	-	-	-	-	-	-	-	1360	-	1357
C-O stretching ester	-	-	-	-	-	-	-	-	-	-	1307
−C-O-C stretching vinyl ether/−C-N-	1263, 1229	1236	1236	1255–1229	1255–1229	1260–1238	1260–1238	1259	1259	1262	1262, 1227
C-N stretching amine	1166, 1154, 1114	1167	1168	1171	1171	1164, 1116	1164, 1116	-	-	-	1151
C-O stretching ester	-	1123	1123	1123	1123	1117	1117	-	-	-	-
C-O stretching ester or C-N	1061	1025	1025	1023	1023	Small broad 1062–1026	1065–1027	1078	1078	1092, 1024	1092, 1062, 1025
C-O-	992	-	-	944	944	Broad 964	978	947	-	-	943
−C=C-	944, 805, 740	947, 728	947, 802, 728	794, 770, 727	770, 731, 802	-	806, 729	803, 757	803, 757	799	799, 740
−C-H bending	916, 865, 827	827	827	-	864	863, 827	865, 827	865, 690	865, 690	864	917, 864
-C=C-	613, 577, 543, 488	629, 606	606, 566, 486	605, 496	605, 570, 486	491	613, 581, 545, 488	599	599	662	662, 613, 575, 544, 487
(**c**)											
Chemical bond	TST 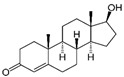	Acrylates						Silicones			
		**A1**	**A1 + API**	**A2**	**A2 + API**	**A3**	**A3 + API**	**S1**	**S1 + API**	**S2**	**S2 + API**
ν (O-H) intermolecular bonded	3411	3533, 3450	3533, 3449	3628, 3542, 3454	3628, 3542, 3454	3454	3454	3485	3610–3250	3473	3428
C-H (CH_2_) stretching alkane	2940, 2871	2960, 2927, 2875, 2857	2960, 2927, 2875, 2857	2953, 2935, 2879, 2863	2953, 2935, 2879, 2863	2958, 2933, 2876	2956, 2927, 2867, 2736	2961, 2902	2961, 2902	2964, 2902	2964, 2902
C=O stretching vinyl/phenyl ether	1662	1737, 1678	1743–1725, 1678	1745–1725	1738, 1676	1737	1737	1853, 1736, 1619	1853, 1768, 1662	-	1661
−C=C- cyclic alkene	1614	-	1617	-	1617	-	1619	-	1613	-	1614
C-H bending alkane methyl group	1467, 1449, 1433, 1417	1460	1460	1462, 1437	1462, 1437	1452	1465–1448	1444	1447	1442	1446
O-H bending alcohol	1375	1373	1373	1372	1372	1378	1378	1412	1412	1412	1416
−C-H- stretching	1351, 1333	-	-	-	-	1334	1334	-	-	1378, 1350, 1330	1375, 1350, 1331
−C-O-C stretching vinyl ether/−C-N-	1272, 1230, 1131, 1056	1238	1244	1255–1229	1239	1260–1234	1260–1234	1254	1254	1259	1259–1230
C-O stretching ester	1189, 1113	1169, 1122	1169, 1122	1171, 1124	1171, 1124	1165, 1116	1116	1189	1189	-	-
−C=C- cyclic alkene	1018, 953, 941, 866, 831	1023, 827	1023, 867, 830	1024, 827	1024, 867	1051, 828,	1051, 867, 828	1076, 843	1076, 843	1089, 1024	1054, 1023
C-O-	956, 941	945	945	944	944	Broad 964	978	948	-	-	956, 941
−C-H bending	778	769, 729	769, 729	770, 728	770, 728	769, 729	769, 739	867, 690, 799, 757	867, 690, 799, 757	926, 864, 797, 702	864, 797, 702
−C=C-	684	630, 605	684, 630, 605	697, 630, 605	684, 630, 605	694	684	690, 599,	690, 599,	685	685
−C-C-	566, 512, 459, 438	491	512, 566	-	512, 566	568, 443	566, 512, 438	-	-	-	-

**Table 4:** Raman identification of IND (b), CYT (a) and TST (c) in the polymeric matrices (peaks or wavenumber ranges underlined are characteristic for API identification in respective patches and not present in placebo polymer film) [19,20,21,22].

(**a**)											
Chemical bond	CYT 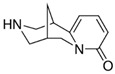	Acrylates						Silicones			
		**A3**	**A3** **+ API**	**A1**	**A1** **+ API**	**A2**	**A2** **+ API**	**S1**	**S1** **+ API**	**S2**	**S2** **+ API**
C=O stretching vinyl/phenyl ether	-	1731	-	1733	-	1734	-	-	-	-	-
N-C=O	1644	-	-	-	-	-	1644	-	-	-	
−C=C- cyclic alkene	1540, 1561	-	-	-	1543	-	1540, 1561	-	1546	-	1561, 1541
C-H bending alkane methyl group	1419, 1468	1450	1457	1449	1460	1445	1419, 1468	-	1470, 1445	1407	1468
O-H bending alcohol	-	1312	1380	1200–1380	1311	1200–1380	-	1412	1416	-	1415
−C-N- stretching	1344	1250–1400	1358	1200–1380	1274	1200–1380	1344	-	-	-	-
-C-O-C stretching vinyl ether/−C-N-	1263, 1223, 1207	-	1218	1200–1380	1210	1200–1380	1263, 1223, 1207	1257	1266, 1226, 1207	1262	1262, 1223, 1210
C-N stretching amine	1153	1154	1165, 1151	1146	1159, 1142	-	1153	-	1152	-	1155
C-O stretching ester	1110	-	-	1115	1107	1123	1110	-	1110,	-	-
C-O stretching ester or C-N	1011, 1035, 1056, 1077, 1092	1060	1035	1041, 1061	1094, 1080, 1062, 1042, 1015	1061, 1020	1011, 1035, 1056, 1077, 1092	-	1087, 1057, 1039, 1011	-	1094, 1078, 1056, 1036, 1014
C-O-	992	-	978	-	983	-	992	-	-	-	978
−C=C-	977, 735	890, 772	806	-	797, 741, 717, 739	895, 877	977, 735	790, 709,	978	793	793
−C-H bending	905, 860, 818	875, 832	838	821, 891	908, 868, 821,	830	905, 860, 818	854	908,	865	905, 864, 818, 790, 737,
−C=C-	716, 655, 613, 574, 539, 511, 485, 455, 376, 352	-	454, 525	625, 766	659, 616, 578, 540, 489, 455, 381, 357	630, 430–330	716, 655, 613, 577, 539, 511, 485, 455, 376, 352	691, 613, 486	714, 656, 615, 575, 485, 377	707,688, 488	715, 614, 575, 539, 488, 378, 354
(**b**)											
Chemical bond	IND 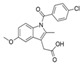	Acrylates						Silicones			
		**A3**	**A3** **+ API**	**A1**	**A1** **+ API**	**A2**	**A2** **+ API**	**S1**	**S1** **+ API**	**S2**	**S2** **+ API**
C=O stretching vinyl/phenyl ester	-	1731	-	-	-	1731	1731	-	1728	-	-
Asymmetric acid -C=O ketone	1698	-	-	-	-	-	-	-	1697	-	1699
(benzoilo) −C=O amide	1620, 1589	-	-	-	-	-	1586	-	1619, 1587	-	1619, 1589
C-H bending alkane methyl group	1467	1450	1456	1457	1447	1446	1443	-	-	-	1469
−C=C-	1438	-	-	1434	-	-	-	-	1438	-	1439
C-H (CH_3)_	1396	-	-	-	-	-	-	1409	1410	1410	1394
O-H bending alcohol	1360	-	1358	1357	1359	1380–1240	1355	-	1360	-	1360
−C-O- acidic group	1311	-	1327	1327	1306	1310	1310	-	1310	-	1310
-C-O-C-	1263, 1221	-	1220	1217	-	-	1223	1265	1265, 1220	1265	1265, 1222
−C-N- or/and C-O stretching ester	1172	1160	1150	-	-	-	-	-	1170	-	1173
	1145	1149	-	1164, 1149	1148	-	-	-	1145	-	1148
O-H alcohol	-	1114	-	-	-	1116, 1120	1125	-	-	-	-
-C-Cl-	1068, 1087	1062	-	-	1060	1064, 1045	1064, 1045, 1086	-	1088	-	1089, 1069
−C-O-C-	1023	1045	1040	1033	-	1022	1022	-	1022	-	1023
C-O stretching primary alcohol	-	997	997	-	-	-	-	-	-	-	1018
C-O vinyl acetate	966	940–980	970	997, 972	-	-	-	-	966	-	967
−C-H-	926, 907	-	926	939, 897	-	-	-	-	908	-	907
strong C-H bending	-	879, 896	835, 896	840, 896	892	892	892	-	-	-	-
−C-Cl- strong C-H bending	840	829	807	805, 838	827	830	830	870–840	839	864	841
C-H bending	756, 738, 701, 630, 399, 271	772	736, 399	526, 452, 395	734, 628	633	630, 698, 737	786 754, 706, 687, 614, 490	755, 738, 700, 615, 487, 431, 411, 397, 271	790, 708, 490	756, 738, 700, 630, 617, 490, 432, 413, 397, 275
(**c**)											
Chemical bond	TST 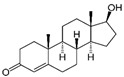	Acrylates						Silicones			
		**A3**	**A3** **+ API**	**A1**	**A1** **+ API**	**A2**	**A2** **+ API**	**S1**	**S1** **+ API**	**S2**	**S2** **+ API**
C=O stretching vinyl/phenyl ether	-	1735	-	1731	-	1734	1731	-	-	-	-
−C=C- cyclic alkene	1654	-	-	-	1663	-	1664	-	1655	-	1652
C-H bending alkane methyl group	1610	-	1611	-	1613	-	1612	-	1612	-	1611
O-H bending alcohol	1450	1454	-	1446	1446	1446	1447	-	-	-	1446
−C-O-C stretching vinyl ether/-C-N-	1349	-	-	-	-	-	-	1409	1410	1411	-
C-O stretching ester	-	-	-	1347	1326	-	-	-	1330	-	1331
−C=C- cyclic alkene	-	1310	-	1308	-	1308	-	-	-	-	1310
C-O-	1281, 1233	-	-	1238	1233	-	1231	1261	1283, 1232	1263	1280, 1233
−C-H bending −C=C-	1205, 1192	1159	-	-	-	-	1195	-	1151	-	1151
	1134	-	-	1143	1153	1120	1129	-	1131	-	1131
−C-C-	1113	1117	-	1118	1115	-	-	-	-	-	-
C-O stretching	1091, 1032	1047	-	1062	-	1063, 1020	1061, 1020, 1086	-	-	-	1091
C=C bending	998, 949, 912, 901, 868	966	-	892, 873, 826,	999, 949, 903, 870, 939	892	1001, 946, 894,	-	997, 947, 902, 869	-	998, 948, 902, 867
C-H bending	839, 746, 717, 681, 662, 541	896, 879, 835, 771	-	766, 628	748, 720, 684, 635, 544	831, 765, 631	829	852, 788, 707, 614, 487	837, 746	863, 790, 709, 490	837, 745, 712, 683

**Table 5:** Melting temperatures of API recorded in thermograms of the tested silicone patches and the effect of API on glass temperatures recorded for acrylic patches.

	Placebo Patch	IND	CYT	TST
Melting temperature T_m_ (°C)				
Pure API		160.9	156.1	154.4
A1, A2, A3		n.d.	n.d.	n.d.
S1		160.0	151.0	152.7
S2		160.1	155.9	153.4
Glass transition temperature T_g_ (°C)				
A1	−26.0	−25.7	−26.7	−27.8
A2	−13.8	−17.6	−17.9	−15.3
A3	−14.3	−18.1	−17.6	−19.6

n.d.—not detected.

## Data Availability

Not applicable.

## Supplementary Materials

The following supporting information can be downloaded at: https://www.mdpi.com/article/10.3390/polym14142888/s1, Figure S1. Infrared spectra of the acrylic patches A1—placebo and drug loaded: (a) IND; (b) CYT; and (c) TST. The grey bands mark the differences or similarities between the spectra of the placebo polymers and API’s characteristic band. Figure S2. Infrared spectra of the acrylic patches A2—placebo and drug loaded: (a) IND; (b) CYT; and (c) TST. The grey bands mark the differences or similarities between the spectra of the placebo polymers and API’s characteristic band. Figure S3. Infrared spectra of the acrylic patches A3—placebo and drug loaded: (a) IND; (b) CYT; and (c) TST. The grey bands mark the differences or similarities between the spectra of the placebo polymers and API’s characteristic band. Figure S4. Infrared spectra of the silicone patches S1—placebo and drug loaded: (a) IND; (b) CYT; and (c) TST. The grey bands mark the differences or similarities between the spectra of the placebo polymers and API’s characteristic band. Figure S5. Infrared spectra of the silicone patches S2—placebo and drug loaded: (a) IND; (b) CYT; and (c) TST. The grey bands mark the differences or similarities between the spectra of the placebo polymers and API’s characteristic band. Figure S6. Raman spectra of polymeric patches with (A) indomethacin; (B) cytisine; and (C) testosterone. Arrows indicate the characteristic API bands also described by rectangular gray stripe. Table S1. The effect of placebo patches on the enthalpy values of endotherms of API.
