# Supplementary material for: Microscopic and Spectroscopic Imaging and Thermal Analysis of Acrylates, Silicones and Active Pharmaceutical Ingredients in Adhesive Transdermal Patches
Source: Polymers (Basel). 2022 Jul 16;14(14):2888. doi: 10.3390/polym14142888 (PMC9322838; doi:10.3390/polym14142888)
Supplement: Supplementary file 1 [file polymers-14-02888-s001.zip › polymers-1755787-SI.pdf]

SUPPLEMENTARY MATERIAL

FTIR spectroscopy

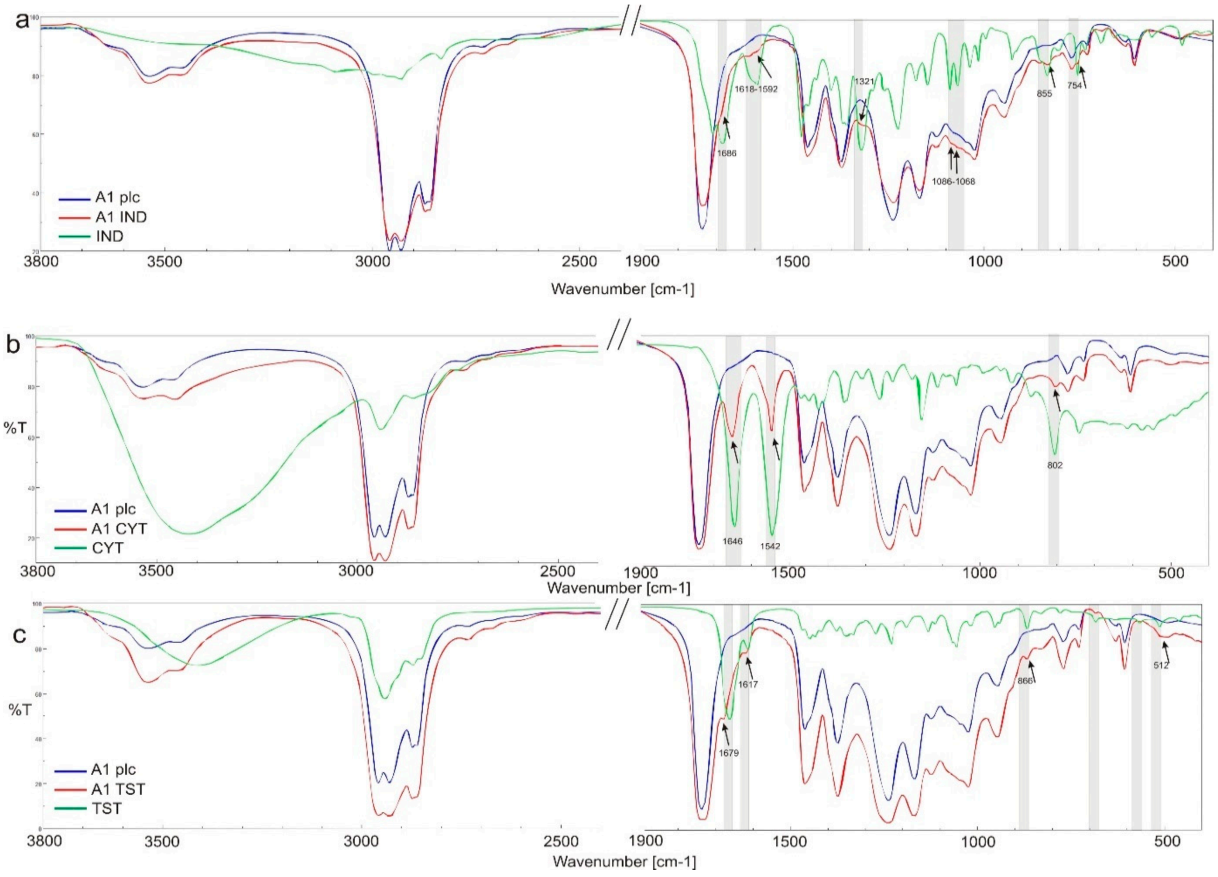

Figure S1. Infrared spectra of the acrylic patches A1 – placebo and drug loaded: a) IND, b) CYT, c) TST. The grey bands mark differences or similarities between spectra of the placebo polymers and API's characteristic band.

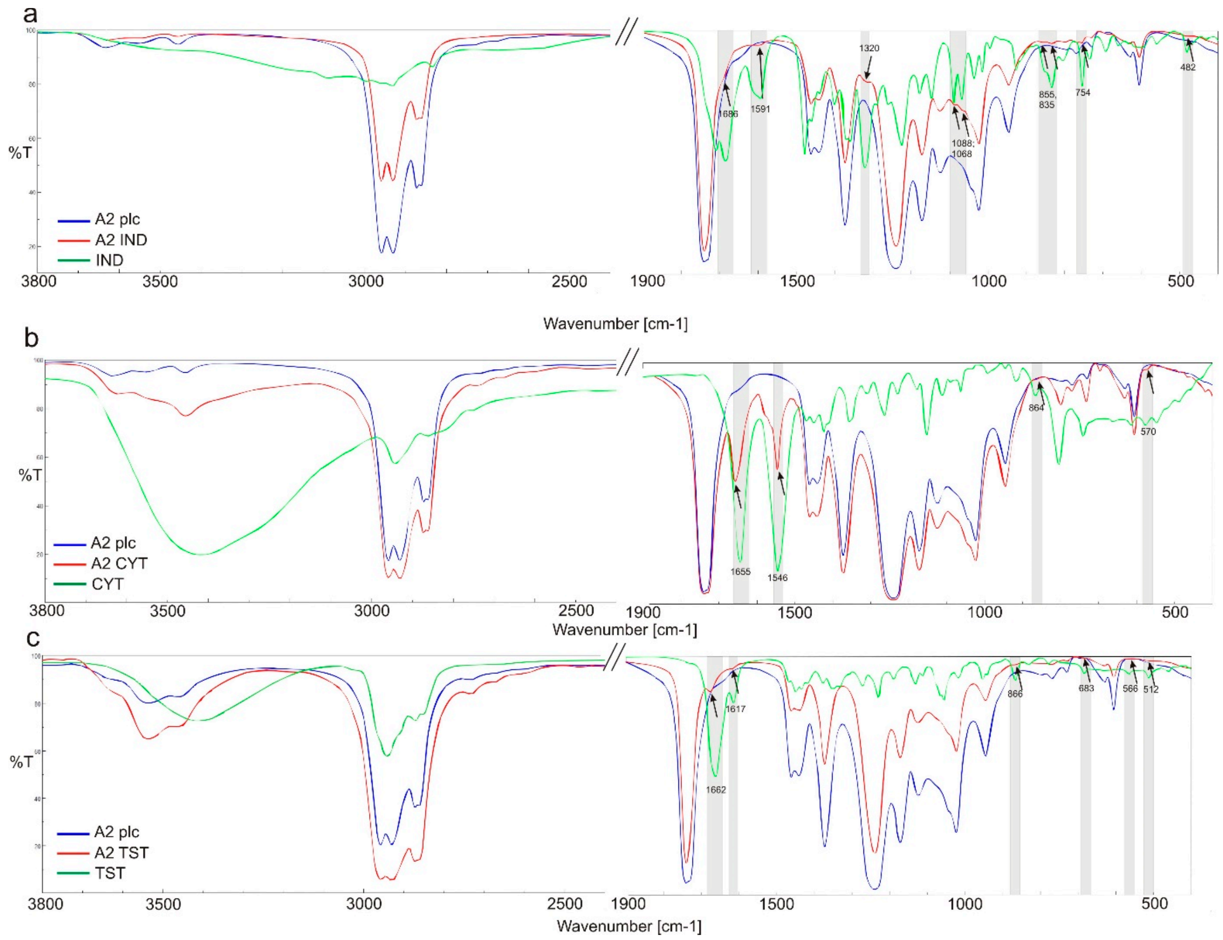

Figure S2. Infrared spectra of the acrylic patches A2 – placebo and drug loaded: a) IND, b) CYT, c) TST. The grey bands mark differences or similarities between spectra of the placebo polymers and API's characteristic band.

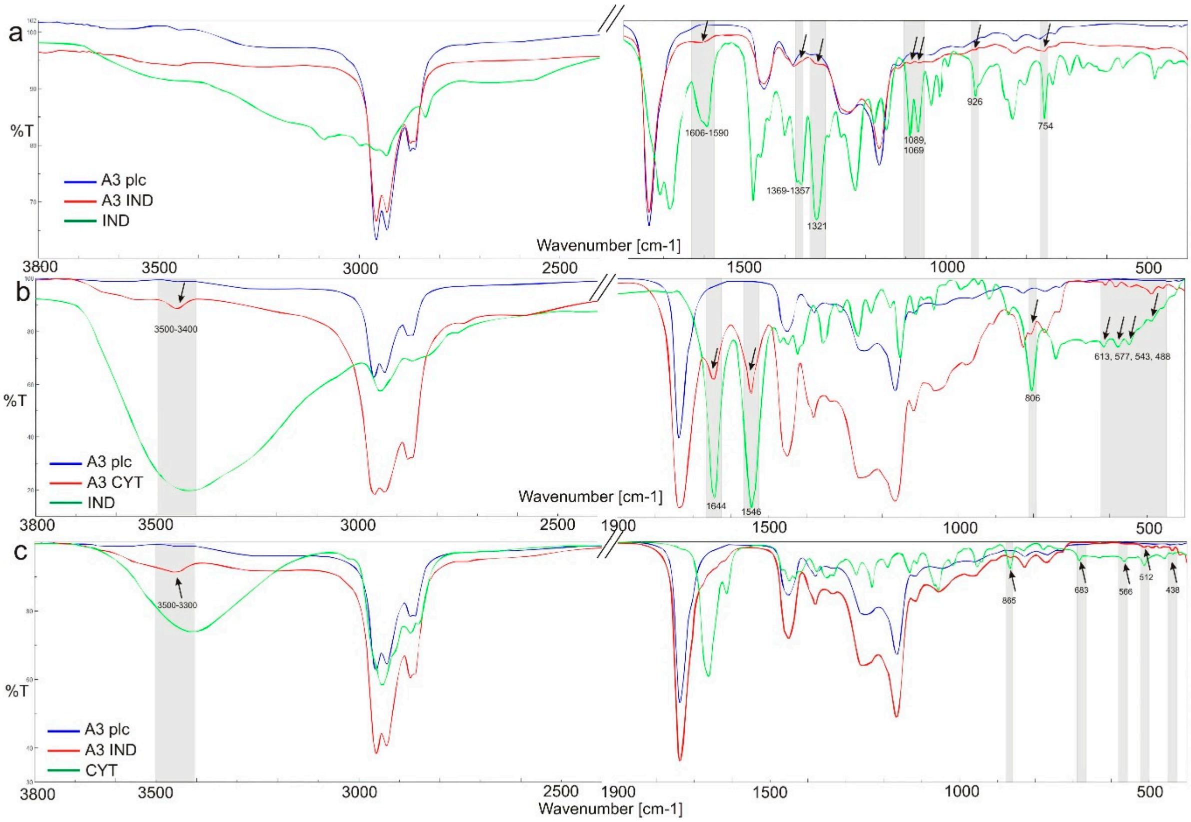

Figure S3. Infrared spectra of the acrylic patches A3 – placebo and drug loaded: a) IND, b) CYT, c) TST. The grey bands mark differences or similarities between spectra of the placebo polymers and API's characteristic band.

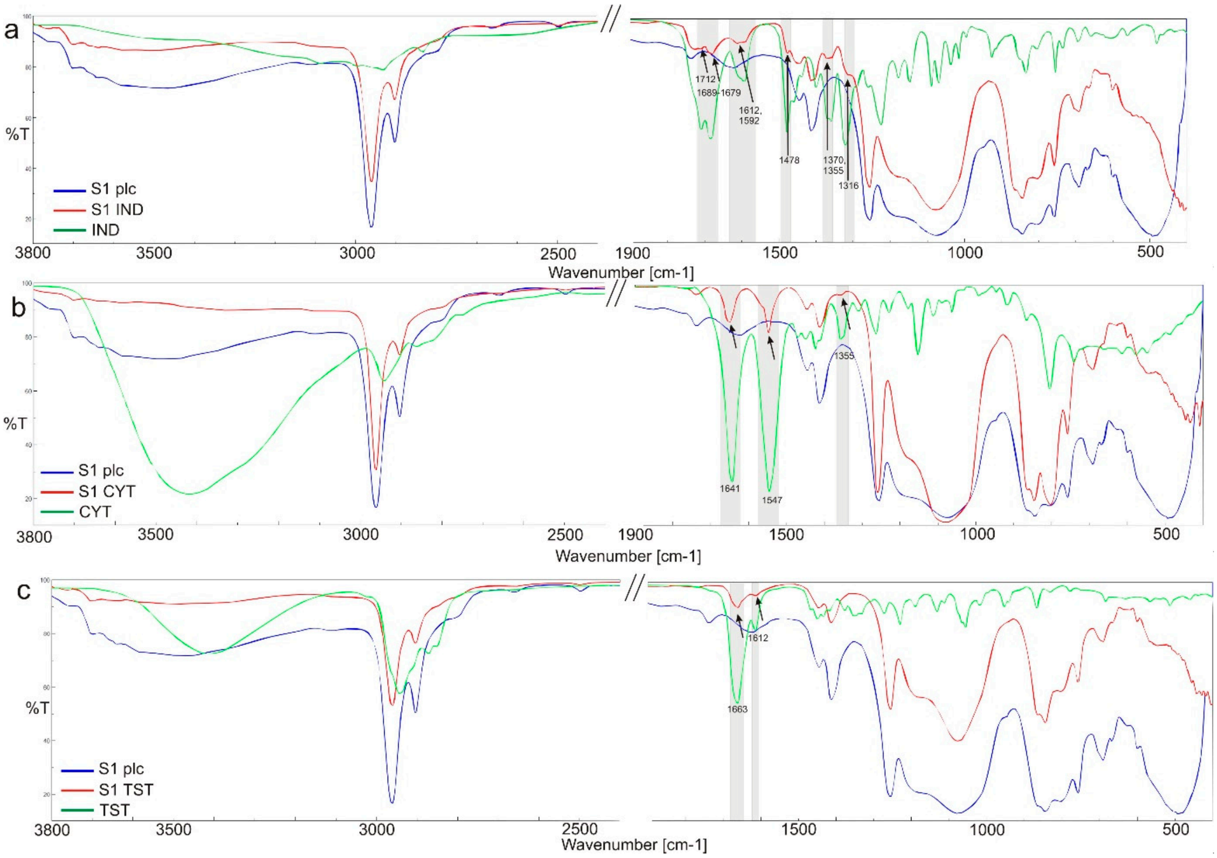

Figure S4. Infrared spectra of the silicone patches S1 – placebo and drug loaded: a) IND, b) CYT, c) TST. The grey bands mark differences or similarities between spectra of the placebo polymers and API's characteristic band.

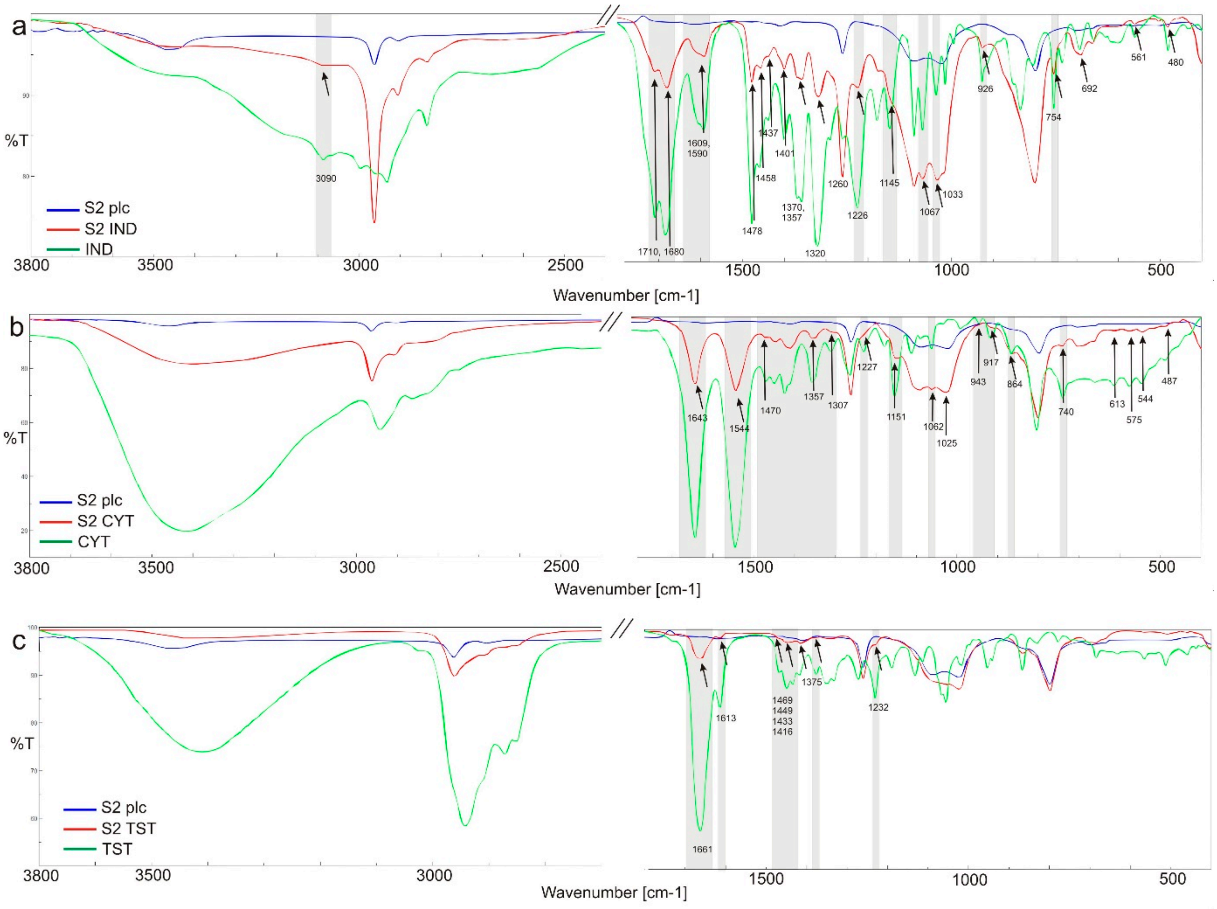

Figure S5. Infrared spectra of the silicone patches S2 – placebo and drug loaded: a) IND, b) CYT, c) TST. The grey bands mark differences or similarities between spectra of the placebo polymers and API's characteristic band.

Raman Spectroscopy

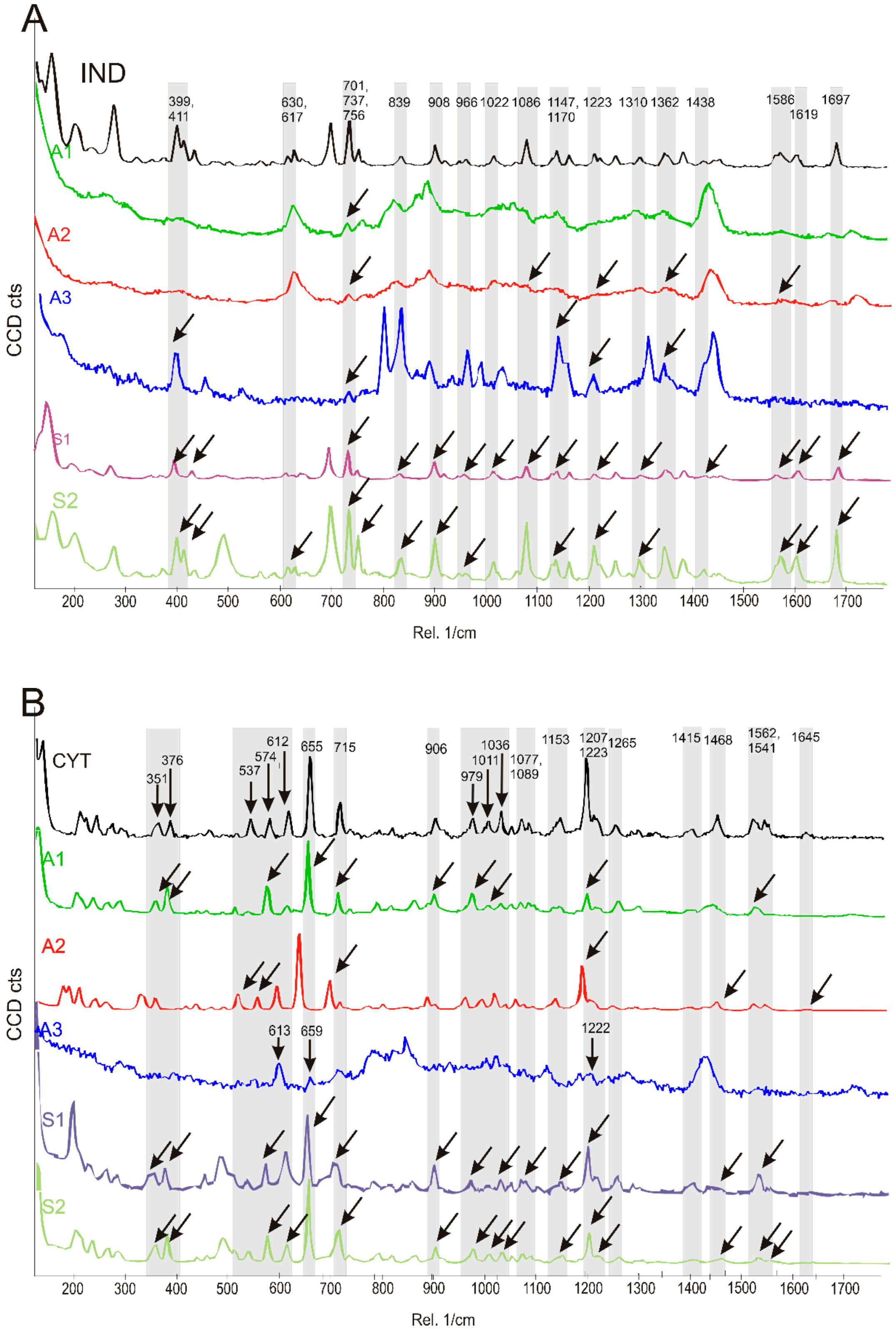

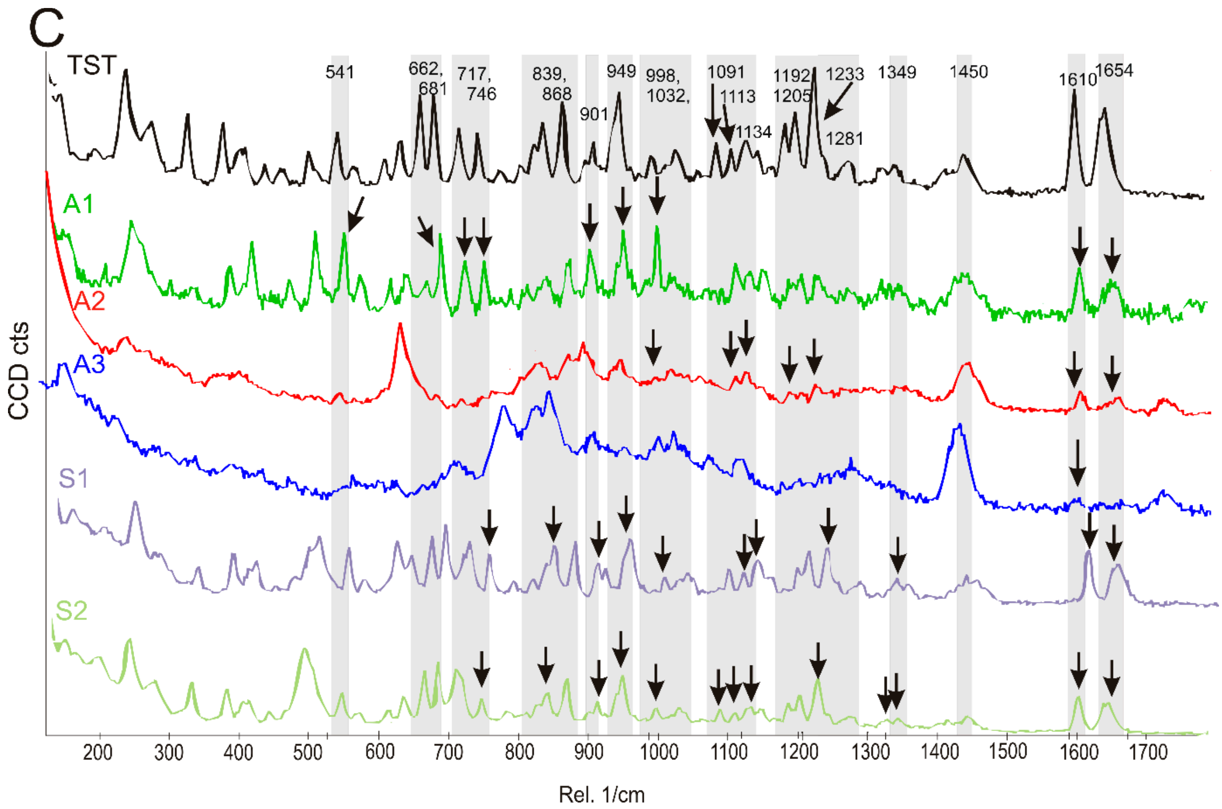

Figure S6. Raman spectra of polymeric patches with A) indomethacin, B) cytisine, C) testosterone. Arrows indicate the characteristic API bands described also rectangular gray stripe.

Table S1. The effect of placebo patches on the enthalpy values of endotherms of API – enthalpy is proportional to the amount of API but 20-50 times smaller than calculated for the corresponding amount of pure API (with no placebo patch placed in the same pan).

API	IND		CYT	
Mass	**0.5 mg**	**1.0 mg**	**0.5 mg**	**1.0 mg**
Pure	-68.9	-106.1	-73.2	-141.6
	in the presence of placebo patch			
A1 (10 mg)			-4.12	-11.78
A2 (10 mg)	-1.3	-2.5	-5.20	-10.78
A3 (10 mg)			-4.08	-12.38
S2 (10 mg)	-3.4	-6.2	-5.79	-11.89
S2 (1 mg)				-58.68
